# Evaluation of a National Online Educational Program in Geriatric Psychiatry

**DOI:** 10.1007/s40596-015-0377-y

**Published:** 2015-06-25

**Authors:** Marcus Law, Mark J. Rapoport, Dallas Seitz, Marla Davidson, Robert Madan, Andrew Wiens

**Affiliations:** 1 University of Toronto, Toronto, ON Canada; 2 Sunnybrook Health Sciences Centre, Toronto, ON Canada; 3 Queen’s University, Kingston, ON Canada; 4 University of Saskatchewan, Saskatoon, SK Canada; 5 University of Ottawa, Ottawa, ON Canada

**Keywords:** Career development, Evaluation, Faculty development, Licensure, Residents, Geropsychiatry

## Abstract

**Objective:**

This study provides evaluation results of an online study group (OSG) for geriatric psychiatry continuing professional development.

**Methods:**

The OSG is an interactive, expert-facilitated, asynchronous educational experience for psychiatrists and residents in Canada. A retrospective web survey assessed self-efficacy, knowledge in geriatric psychiatry, comfort with online learning, and perceived effectiveness of the instructional methods. Wilcoxon signed-rank tests and descriptive statistics were calculated.

**Results:**

Twenty-nine (of 50) participants (58 %) completed the questionnaire. Although only 48 % of respondents reported improved perceived knowledge, 79 % reported improved efficacy beliefs, and 76 % reported improved comfort with online learning. Most (79 %) would consider taking OSG again, and 93 % would recommend it to others.

**Conclusions:**

The OSG was well-received, with greater benefits for self-efficacy with the material and comfort with online learning than for perceived knowledge itself. Further research is needed to ascertain actual knowledge change in the context of online learning in medical education.

The growth of an aging population requiring more effective interventions to improve mental health and well-being has highlighted the need for continuing professional development (CPD) in geriatric psychiatry for general and geriatric psychiatrists [1]. With the recent formal recognition of geriatric psychiatry as a subspecialty designation in Canada, those practicing in this area are required to demonstrate competence through a new certification examination. At the time this manuscript was written, only five Canadian universities offered accredited subspecialty residency positions in geriatric psychiatry, producing few certified specialists. Of the 4970 psychiatrists in Canada, only 4.8 % were certified in geriatric psychiatry as of October 2014, although 7.6 % are mainly practicing geriatric psychiatry [2].

CPD is crucial in maintaining physicians’ competence and social accountability, allowing them to meet the needs of the populations they serve [3], and represents one strategy to prepare physicians for the certification exam. However, deficiencies exist in the design of CPD programs: (a) they are typically less academically rigorous than a residency program; (b) they generally deliver content over a short period; and (c) knowledge gained through traditional CPD seldom leads to sustained changes in practice [4]. Internet-based technologies offer new CPD opportunities that address barriers such as time, distance, and distribution of content to many users, anytime, anywhere [5]. Online discussions help achieve learning objectives through instructor-student and student-student interactions [6].

This study provides a critical analysis of an innovative online model for geriatric psychiatry CPD. Evaluation results are reported with a discussion of implications for online learning in medical education.

## Methods

### Program Overview

The Royal College of Physicians and Surgeons of Canada (RCPSC) approved geriatric psychiatry as a subspecialty in 2009, with the first certification examination held in September 2013. Residents require two years’ training followed by a subspecialty examination, and practicing psychiatrists who care for the elderly are not exempt from the examination. We established an online study group (OSG) to reinforce and consolidate learning, assist with examination preparation, and address knowledge limitations and inadequate engagement in traditional lecture-based learning. The first OSG cohort participated from October 2012 to August 2013.

### Curriculum Design

The OSG is an interactive, expert-facilitated, asynchronous study group. A needs assessment was undertaken to plan the program, involving (1) discussion of the need for such programs at the Canadian Academy of Geriatric Psychiatry (CAGP) Board Strategic Planning Meeting; (2) review of RCPSC official objectives; (3) survey of CAGP members (*n* = 65 geriatric psychiatrists, family physicians, geriatricians); and (4) review of needs assessment data and selection of topics and facilitators.

Initially, there were five study groups with ten participants each, balanced in years in practice, geography, and academic/community settings. Midway throughout the 2012–2013 year, the five study groups were consolidated into three, as some groups were not as active in the online discussion. The groups were, therefore, collapsed so as to have slightly larger groups to generate more discussion within each group.

There were 22 distinct modules, each lasting 14 days. Modules focused on: geriatric psychiatry (e.g., anticholinergic drugs and inappropriate medications; primary psychotic disorders; sleep difficulties and disorders; anxiety disorders; pharmacotherapy of depression; non-pharmacological treatment of neuropsychiatric symptoms of dementia; aging and psychopharmacology; epidemiology), palliative care in geriatric psychiatry, psychosocial issues (e.g., consent, elder abuse, caregiver distress, care in nursing home and community outreach settings), and psychotherapy (e.g., dynamic therapy with bereavement and other non-expert roles of geriatric psychiatrists; cognitive behavioral, interpersonal and group therapy). A final module entitled “Other Topics” incorporated suggestions of the group and allowed an opportunity to discuss with peers and the organizing group other topics or questions not already covered. For each module, a facilitator (i.e., geriatric psychiatrist) recommended two current review papers and one primary research paper in geriatric psychiatry. After completing assigned readings, participants logged into the portal to participate in four different discussion boards or “rooms” within their “group page.” The first room contained short-answer questions covering essential elements of the module’s topic. The second focused on reflection and discussion of broader concepts and controversies. The third was a journal club to critically appraise a topical empirical paper. In the fourth, “Clinical Corner,” participants discussed a challenging case provided by the facilitator or themselves, or asked the facilitator and their peers questions about topics they have “always-wanted-to-know-about-but-were-afraid-to-ask.” Audio or video recordings and electronic presentations from a recent parallel didactic lecture series were available to participants. Over time, in response to feedback, the faculty began to offer “official answers” at the end of each module. References were provided prior to each module to allow more preparation time. Institutional research ethics board granted ethics approval.

### Study Design and Data Analysis

Members of the CAGP's other partner organizations were invited by email to participate in the OSG. The 2012–2013 inaugural program was evaluated using (a) retrospective post-then-pre design to allow participants to reflect on what they learned, thus reducing the response shift bias that is associated with self-report measures, and (b) post-test only design. Data were collected using a web-based survey administered to all participants. The retrospective post-then-pre-design survey assessed program effects in three key domains: (a) self-efficacy (participants’ confidence in their ability to pass the geriatric psychiatry exam), (b) knowledge in geriatric psychiatry (participants’ perceived knowledge of the assessment and treatment of geriatric psychiatric disorders), and (c) comfort level with online learning. Each domain was measured using three to six items, rated on a five-point Likert scale (strongly disagree, disagree, neither agree nor disagree, agree, strongly agree). Analysis of items within the domains demonstrated adequate reliability (Cronbach’s *α* = 0.70[a]; *α* = 0.92[b]; *α* = 0.73[c]). Wilcoxon signed-rank tests were calculated.

Self-report post-test only questions assessed participants’ perceptions of the effectiveness of instructional methods (i.e., short-answer questions, reflective questions, clinical corner cases, journal club, active participation) to improve their understanding of the subject matter. Each method was measured by a single question using a five-point Likert scale (not at all, somewhat, moderately, mostly, a great deal). Descriptive statistics (proportions) were calculated.

Responses to open-ended questions about program quality provided additional detail about perceived effectiveness of instructional methods. Data were analyzed using thematic analysis to identify patterns within the data. Coded data were grouped into themes to capture its critical aspects in relation to the topics of interest. Quotations considered to be exemplary representations of the themes were selected for this manuscript.

## Results

### Participant Description

Forty-five practicing geriatric psychiatrists and five geriatric psychiatry trainees participated in the 2012–2013 OSG (Table 1). Most practicing psychiatrists and residents were female (64.6 and 80.0 %, respectively). There was a broad distribution in number of years of experience practicing psychiatrists had at time of enrollment (mean = 14.35; SD = 9.86). Participating psychiatrists had varied clinical practice backgrounds, most with elements of hospital-based (66.6 %) or community-based practices (55.5 %).

**Table 1:** Characteristics of 2012–2013 online study group participants

Participant category	Characteristic	Value
Practicing psychiatrists (*n* = 45)	Sex, *n* (%)	
	Female	29 (64.4)
	Male	16 (35.6)
	Years in practice	
	Mean (SD)	14.35 (9.86)
	Median (IQR)	13 (7–21)
	0–5 years, *n* (%)	7 (15.5)
	6–10 years	9 (20.0)
	11–15 years	6 (13.3)
	16–20 years	7 (15.5)
	>20 years	10 (22.2)
	Missing	6 (13.3)
	Practice setting, *n* (%)	
	Academic	20 (44.4)
	Hospital	30 (66.6)
	Community	24 (55.5)
	Long-term care	6 (13.3)
	Missing	5 (11.1)
Residents (*n* = 5)	Sex, *n* (%)	
	Female	4 (80.0)
	Male	1 (20.0)

The web-based survey was completed by 29 (of 50) participants, a response rate of 58 %. There were no differences between respondents and non-respondents with respect to gender (chi-square (1,49) = 1.27, *p* = 0.261) or years in practice (t(1,41) = 1.79, *p* = .188). A significant positive effect was observed in each of two domains: 79 % (*n* = 23) of survey respondents reported improved efficacy beliefs (*z* = −3.69, *p* < .001); 76 % (*n* = 22) reported improved comfort with online learning (*z* = −3.75, *p* < 0.001 (Table 2). Conversely, only 48 % (*n* = 14) reported improved perceived knowledge of geriatric psychiatry (*z* = −2.12, *p* < 0.05), 41 % (*n* = 12) noted there was no change, and 10 % (*n* = 3) suggested that the OSG had a negative impact on their perceived knowledge of psychiatry (Table 2).

**Table 2:** Survey respondents’ reports on the effects of the online study groups (*n* = 29)

	*n* (%)			Wilcoxon signed-rank test
	Positive impact	No change	Negative impact	
Efficacy beliefs	23 (79.31)	4 (13.79)	2 (6.90)	*z* = −3.69, *p* < 0.001
Perceived knowledge of geriatric psychiatry	14 (48.28)	12 (41.38)	3 (10.34)	*z* = −2.12, *p* < 0.05
Comfort with online learning	22 (75.86)	4 (13.79)	3 (10.34)	*z* = −3.75, *p* < 0.001

Most respondents had at least moderately positive perceptions of the effectiveness of the five pedagogical approaches to improve their understanding of the subject matter, namely short-answer questions (*n* = 26; 90 %), reflective questions (*n* = 24; 83 %), clinical corner cases (*n* = 22; 76 %), active participation (*n* = 21; 72 %), and journal club (*n* = 16; 55 %). The level of positive perceptions varied across approaches, with short-answer questions being most popular and journal club being least popular (17 % stated it was not at all effective). Approximately half rated all approaches, except journal club, as having improved their understanding of the subject matter “mostly” or a “great deal”. Additionally, 79 % (*n* = 23) reported they would consider taking the program again, and 93 % (*n* = 27) specified they would recommend the program to others.

## Discussion

The OSG was well-received, with benefits of improved efficacy beliefs and comfort with online learning for most participants, and to a much lesser extent, perceived knowledge of geriatric psychiatry. In e-Learning for health professionals, interactivity, practice exercises, and feedback have been demonstrated to improve learning outcomes, while satisfaction increases with interactivity and online discussion [7]. These reflect the OSG’s pedagogical approaches.

While it is known that well-structured journal clubs can be stimulating and educational [8], the journal club was rated poorly by respondents. Further research is needed to identify factors influencing uptake, especially in the context of CPD with a diverse group of participants with varied expertise, experience, and learning style preferences. It would be interesting to see whether participants prefer a learning format consistent with the final assessment design—in our case, the certification examination is in short-answer question format, which may have reflected participants’ preferred learning format.

Three-quarters reported increased feelings of self-efficacy in terms of passing the certification exam and increased comfort with online learning. Given the trend towards e-Learning and advantages technology can offer for CPD, this initiative is timely. Benefits of e-Learning include flexibility through 24-h access, allowing individuals to set the pace and timing for learning [5], improved accessibility for isolated practitioners [5], reduced time spent traveling to lectures [5], standardized teaching materials [5], and interactive learning [7].

While efficacy beliefs and comfort with online learning improved for most and the majority would consider taking the OSG again or recommending it to others, it is noteworthy that less than half reported improved perceived knowledge of geriatric psychiatry, and 41 % of participants reported no change in their perceived knowledge of geriatric psychiatry. A further 10 % stated that the OSG had a negative impact on their perceived knowledge. The reason for this discrepancy is unclear, but assessment of actual knowledge using a pre- and post-test study design may be helpful in understanding participants’ true increase/decrease in knowledge. As Eva and Regehr [9] noted, “humans do not self-assess well”; they suggest that we should not rely on self-assessments to provide valid indications of knowledge/ability. One might speculate that efficacy beliefs might serve as a mediating factor and additionally contribute to knowledge uptake and future practice improvement. Alternately, for some participants, the course may have highlighted gaps in their knowledge of which they were previously unaware. As this study did not assess actual knowledge change, it would be interesting to determine whether actual knowledge acquisition parallels or diverges from perceived knowledge change, as evaluated in this study.

As a new generation of learners becomes practicing physicians, they will continue to demand online CPD programs to suit their schedules, lifestyles, and learning needs. Hence, future research should transition towards a better understanding of the ways in which online CPD education can be made successful for a diverse group of participants, shifting away from an emphasis on comparing the effectiveness of online delivery versus traditional methods to instead focus on when to use e-Learning, how to blend it with traditional methods or other emergent approaches (e.g., social media), and how to use it to achieve specific learning objectives and its cost-effectiveness [5]. Lastly, given the peer group model employed, a point of interest is whether active participation would enhance the effectiveness of the OSG. Hrastinski said that “online learner participation is a process of learning by taking part and maintaining relations with others. It is a complex process comprising doing, communicating, feeling and belonging, which occurs both online and offline” [10, p.1761]; this emphasizes that participation should move beyond writing to doing and communicating. Individuals in this e-Learning initiative referred to participation as taking part and joining in a dialogue. The self-accredited nature of the course and the lack of mandated participation may be barriers to more active participation.

Future research should further clarify when specific online designs are indicated and how to use them effectively [6]. The next steps may include analysis of data from subsequent online CPD courses and the addition of an objective outcome measure, including knowledge. Others have also highlighted a need for assessment of whether educational initiative impacts skills and behaviors in practice, and effects on patient care [7]. The retrospective design and response rate of 64 % may be limitations of the current report.

Overall, the evaluation data provide evidence that most participants perceived value in the OSG and found it to be useful to help them prepare for the RCPSC geriatric psychiatry exam, increase their perceived knowledge of the content material and enhance their comfort with online learning. Attention has been directed toward participants’ suggestions for improvement to ensure a stronger offering in subsequent iterations. A second cohort, largely of new participants, participated from November 2013 to August 2014 with changes that included consolidating participants into one large study group, dropping the less popular journal club, adding facilitators, making references more easily accessible and manageable in length, and providing reference lists for each module 2 weeks in advance. Further evaluation is needed to ascertain possible modifications to this program and other e-Learning methods that could contribute to an improvement in perceived or actual knowledge, as well as furthering interactivity when participation is not mandatory.

**Table:** 

Implications for Educators
• An interactive, expert-facilitated, asynchronous online study group (OSG) for psychiatrists and residents may have benefits for many participants (improved efficacy beliefs, comfort with online learning and, to a much lesser extent, perceived knowledge of geriatric psychiatry).
• Caution is warranted as not all survey respondents report improvements in perceived knowledge of geriatric psychiatry; although most respondents reported they would consider taking the program again would recommend the programs to others.
• Further research is needed to ascertain actual knowledge change in the context of online learning in medical education, and optimal ways of facilitating interactivity when online active participation is not mandatory.
